# Sanitary Code for Texas

**Published:** 1910-02

**Authors:** 


### Abstracts and Selections.

### Sanitary Code for Texas.

Synopsis of Sanitary Code for Texas, adopted by the Texas State Board of Health and approved by the Governor. Becomes operative February 1, 1910, and has the absolute force of law. Any person violating any of its rules and regulations are guilty of a misdemeanor and on conviction liable to a penalty of not less than $10 nor more than $1000.

### QUARANTINE AND DISINFECTION.

The new rules on the subject of the management of contagious diseases are clear and concise, but vary very little from those previously in vogue. All cases of a contagious nature are required to be placed under restrictions by the first doctor that sees them, and the attending physician is given authority to do this. He next notifies the local health officer, and, in case of pestilential disease, notifies the President of the State Board of Health by wire.

### DISEASES REPORTABLE.

The reportable diseases include Asiatic cholera, bubonic plague, typhus, yellow fever, smallpox, scarlet fever, diphtheria, epidemic dysentery, trachoma, tuberculosis and anthrax, and report made monthly to the State Board of Health.

Several kinds or degrees of disinfection, isolation and quarantine are described for the various diseases, and local health officers, attending physicians, school officials and others are required to establish the various kinds of quarantine and isolation. With smallpox, diphtheria, scarlet fever or other quarantinable diseases, house must be placarded and modified quarantine established. The local authorities may, however, exercise additional precautions when they desire, and the above rules do not abrogate their right to do so.

### REGISTRATION OF THE TUBERCULAR COMPULSORY.

It will be observed that consumption is reportable. The reports on tuberculosis are to be privately kept and are to be considered in the light of a confidential communication not for the purpose of isolation, but with the object of education in sanitary precautions, and to supply literature of the State Board of Health. Infected house vacated because of death or removal must not be occupied until disinfected. In case any infected house or premises are not promptly disinfected, the local health officer shall place on the house a placard warning the public not to occupy or enter said house until it is disinfected. This is also required of houses or apartments previously occupied by consumptives. All families in which a case of smallpox, diphtheria, scarlet fever, typhoid fever or tuberculosis occurs will be supplied with printed instructions through the health officer or attending physician as how to avoid the spread of the disease.

### BLINDNESS PREVENTED.

Nurses and midwives shall report all cases of eye trouble in the new-born to the local health officer, or, in his absence, to some other physician. Persons suffering from contagious eye diseases, such as granulated lids or trachoma, must be excluded from school and public assemblages until the active stage subsides and they can present a physician's certificate to the effect that they are no longer a menace to others.

VITAL STATISTICS—REGISTRATION BUREAU IN ALL INCORPORATED TOWNS.

Same radical changes are made in the old law on collection of vital statistics. The clerk of court is still the registrar of each county outside of incorporated cities and towns, and such reports of births and deaths are made direct to him. Every incorporated city and town in the State is made a primary registration district, and all births and deaths occurring in such city are to be reported to the city health officer or other city official who acts as city registrar. This is a practice already in the larger cities of the State.

UNDERTAKERS TO MAKE RETURN OF A DEATH.

A new departure is placing the responsibility of reporting deaths upon the undertaker, as is done in other States most successful in collecting such statistics. A burial permit is required in each incorporated city and town, which is issued by the city registrar, but in the rural districts no permit is required, and the certificate of death is sent by the undertaker direct to the clerk of the county court. Physicians, midwives or parents are required to report births. A penalty is provided for that will cause all concerned to take notice.

DEPOTS, RAILWAY COACHES AND SLEEPING CARS.

These rules conform very closely to previous rules on sanitation of public buildings, railway coaches and sleeping cars. The Legislature took away from the Board of Health the right to include public buildings in this Code, but placed same under the requirements of the Advisory Code instead, thereby necessitating the approval of city councils and commissioners courts before public buildings can properly come under the law.

Persons suffering with contagious diseases, including measles and whooping-cough, are prohibited from riding in passenger cars, interurban cars or street cars.

SANITARY REQUIREMENTS.

Waiting rooms in depots and railway coaches, sleeping cars and interurban cars must be properly ventilated and if necessary heated; good and wholesome water furnished patrons and the traveling public; the water cooler must be easily moved and must be emptied, rinsed and cleaned daily; ice must be washed, and must be handled with tongs and not dumped on floors and platforms; it is required that adequate urinals and water closets be provided and that same be kept in sanitary condition; dusting and dry sweeping prohibited at all times; cuspidors must be provided in adequate numbers; expectorating on floors of any above mentioned buildings or cars, including street cars, is made an offense, and brushing of teeth or expectorating in basins used for lavatory purposes prohibited, and placards calling attention to both are required. Premises of depots and stations must be drained and cisterns or water containers screened against the mosquito.

NEGRO PORTERS PLACED UNDER THE BAN.

Negro porters are prohibited from sleeping in berths or using bedding intended for white passengers. Separate compartments are required. This will necessitate berths in the smoking apartments being set aside for their uses.

Buffets and dining cars have the same requirements as to cleaning, with special requirements as to cleaning of refrigerators, food boxes, etc. Sleeping cars are required to be thoroughly cleaned and disinfected twice a week.

Interurban cars and street cars must be washed with a hose and scrubbed thoroughly every day.

A premium is placed upon the vacuum process of cleaning by authorizing a less seldom cleaning, scrubbing or disinfecting when this cleaning apparatus is used.

GOVERNING THE PREPARATION FOR TRANSPORTATION OF DEAD BODIES.

These rules are practically the same as now in use in every State in the Union.

Bodies dead from Asiatic cholera, bubonic plague, typhus fever or smallpox are prohibited from being transported.

Bodies dead from diphtheria, scarlet fever, glanders, anthrax or leprosy must be thoroughly embalmed before shipment.

Bodies dead from diseases which are not contagious may be shipped in a sound coffin or enclosed in a sound wooden box, without other special preparation, provided they will reach their destination within thirty hours from time of death.

Disinterred bodies to be treated as infectious or dangerous and not to be accepted for transportation unless removal has been approved by State and local authorities.

